# Cerebrospinal fluid B cells and disease progression in multiple sclerosis - A longitudinal prospective study

**DOI:** 10.1371/journal.pone.0182462

**Published:** 2017-08-04

**Authors:** Sebastian Wurth, Bettina Kuenz, Gabriel Bsteh, Rainer Ehling, Franziska Di Pauli, Harald Hegen, Michael Auer, Viktoria Gredler, Florian Deisenhammer, Markus Reindl, Thomas Berger

**Affiliations:** 1 Clinical Department of Neurology, Medical University of Innsbruck, Innsbruck, Tirol, Austria; 2 Department of Neurology, Clinic for Rehabilitation Münster, Münster, Tirol, Austria; National Institutes of Health, UNITED STATES

## Abstract

**Background:**

There is evidence that B cells play an important role in disease pathology of multiple sclerosis (MS). The aim of this prospective observational study was to determine the predictive value of cerebrospinal fluid (CSF) B cell subtypes in disease evolution of patients with MS.

**Materials and methods:**

128 patients were included between 2004 and 2012. Median follow up time was 7.9 years (range 3.3–10.8 years). 10 patients were lost to follow-up. 32 clinically isolated syndrome- (CIS), 25 relapsing remitting MS- (RRMS), 2 secondary progressive MS- (SPMS) and 9 primary progressive MS- (PPMS) patients were included. The control group consisted of 40 patients with other neurological diseases (OND). CSF samples were analyzed for routine diagnostic parameters. B cell phenotypes were characterized by flow cytometry using CD19 and CD138 specific antibodies. Standardized baseline brain MRI was conducted at the time of diagnostic lumbar puncture. Main outcome variables were likelihood of progressive disease course, EDSS progression, conversion to clinical definite MS (CDMS) and relapse rate.

**Results:**

CSF mature B cells (CD19+CD138-) were increased in bout-onset MS compared to PPMS (p<0.05) and OND (p<0.001), whereas plasma blasts (CD19+CD138+) were increased in bout-onset MS (p<0.001) and PPMS (p<0.05) compared to OND. CSF B cells did not predict a progressive disease course, EDSS progression, an increased relapse rate or the conversion to CDMS. Likelihood of progressive disease course (p<0.05) and EDSS (p<0.01) was predicted by higher age at baseline, whereas conversion to CDMS was predicted by a lower age at onset (p<0.01) and the presence of ≥9 MRI T2 lesions (p<0.05).

**Conclusion:**

We detected significant differences in the CSF B cell subsets between different clinical MS subtypes and OND patients. CSF B cells were neither predictive for disease and EDSS progression nor conversion to CDMS after a CIS.

## Introduction

Multiple Sclerosis (MS) is an inflammatory demyelinating disease affecting the central nervous system (CNS). Inflammation in MS involves especially T cells, B cells, macrophages, antibodies and cytokines and numerous other immune components [1,2].

B cells play an important role in MS but the extent of its contribution to pathogenesis and progression is still under investigation. Intrathecal immunoglobulin (Ig) synthesis and oligoclonal bands (OCB) are present in the majority of MS patients [3,4]. The presence of OCB within the cerebrospinal fluid (CSF) of MS patients indicates an intrathecally ongoing immune process. The overlap of the Ig transcriptom of B cells with the Ig proteome of peptides in the CSF indicates that B cells produce OCB [5]. The major part of these OCB is typically of the IgG isotype [5–7]. B cell counts are increased in the CSF of patients with clinically isolated syndrome (CIS), MS and other inflammatory neurological disease [8,9]. They persist within the CSF and the CNS [7,10]. Some of the currently available treatments lead to a reduction of lymphocyte numbers, specifically T cells and B cells within the CSF. This reduction is suggested to be associated with treatment benefits on MS disease activity [6]. The outcome of patients treated with B cell targeting antibodies such as rituximab [11], ocrelizumab [12,13] and ofatumumab [14] underlines the likelihood of B cell involvement in the pathomechanisms of MS. These anti-CD20 antibodies deplete B cells, while OCB persist within the CSF. Because of the stable OCB pattern in the CSF of MS patients despite depletion of CD20 positive B cells, plasma cells, which are not targeted by anti-CD20 antibodies, are supposed to persist within the CNS of MS patients and continue producing immunoglobulins [6,15,16]. The role of CSF B cells in MS disease progression is still unclear. Different B cell subsets, such as long-lived plasma cells that do not express CD20, are thought to be involved especially in progressive forms of MS. The presence of CSF B cells in relapsing-remitting MS (RRMS) is well investigated. The main B cell subset that can be detected within the CSF, especially in patients with relapsing onset of MS, are short lived plasmablasts [16–18].

The aim of this prospective study was to analyze CSF B cell phenotypes from patients with clinically isolated syndrome (CIS), relapsing-remitting and chronic progressive (CP) MS by following a previously published cohort long term [4]. We were aiming to further investigate certain B cell phenotypes in the CSF of those subgroups, whether different B cell subpopulations correlate with different MS disease courses and markers such as magnetic resonance imaging (MRI) characteristics, and whether we may identify subgroups of patients that could be eligible and may benefit from a B cell focused treatment [4]. In the follow up analysis we were aiming to find out whether the CSF B cell populations can be used as prognostic markers in the course of disease in MS patients and patients with CIS.

## Materials and methods

### Patients

MS patients and neurological controls were recruited prospectively from 2004 to 2012 at the Clinical Department of Neurology, Medical University of Innsbruck, Austria. This study was approved by the ethical committee of the Medical University of Innsbruck (study Nr. UN2045, 217/4.12, 07.07.2004) and all patients gave written informed consent.

The following inclusion criteria had to be fulfilled by the group of CIS and MS patients: (1) CIS/MS according to the revised McDonald Criteria 2005 or 2010 [19,20], (2) presence of intrathecal immunoglobulin synthesis (elevated Ig-indices and/or oligoclonal IgG bands) [21]. 32 patients with a CIS, 25 patients with RRMS, 2 patients with secondary progressive (SP) MS and 9 patients with primary progressive (PP) MS were included. 10 patients were lost to follow up. All MS patients were examined at the center by a neurologist, including assessment of the expanded disability status scale (EDSS) [22] and confirmation of relapse/disease progression during the follow-up period. Demographic, clinical and CSF characteristics of all MS patients and neurological controls are shown in Table 1.

**Table 1:** Demographic, CSF and clinical data at sampling and follow-up.

	CIS	RRMS	SPMS	PPMS	OND
Number included	40	27	2	9	40
Lost to follow-up	8	2	0	0	0
Finally analyzed	32	25	2	9	40
Females	22 (69%)	18 (72%)	1 (50%)	4 (44%)	26 (65%)
Age at baseline (years) ^1^	27.7 (24.5, 30.9)	33.5 (29.3, 37.7)	36.3, 56.5	50.7 (44.8, 56.6)	38.2 (33.2, 43.3)
CSF CD3+ cells (%) ^1^	89.5 (87.6, 91.4)	90.3 (88.3, 92.3)	86.7, 87.6	91.5 (88.7, 94.2)	91.7 (89.8, 93.7)
CSF CD19+CD138- cells (%) ^1^	4.3 (3.4, 5.3)	3.4 (2.6, 4.2)	5.5, 6.2	1.7 (0.7, 2.7)	0.9 (0.7, 1.2)
CSF CD19+CD138+ cells (%) ^1^	1.5 (1.0, 2.1)	1.6 (0.9, 2.3)	2.2, 2.7	0.7 (0.3, 1.2)	0.4 (0.2, 0.6)
CSF CD19-CD138+ cells (%) ^1^	0.3 (0.2, 0.4)	0.2 (0.2, 0.3)	0.1, 0.3	0.3 (0, 0.7)	0.3 (0.1, 0.4)
CSF CD3-CD19-CD138- cells (%) ^1^	4.3 (2.7, 6.0)	4.4 (3.2, 5.7)	3.5, 5.3	5.8 (3.5, 8.0)	6.7 (4.8, 8.6)
CSF leukocytes / μl ^1^	14.9 (10.0, 19.7)	8.9 (6.0, 11.9)	5.0, 7.3	4.7 (2.3, 7.1)	13.2 (0.3, 26.1)
CSF IgG OCB positive	30 (94%)	24 (96%)	2 / 2	8 (89%)	5/36 (14%)
IgG-index ^1^	1.1 (0.9, 1.4)	1.1 (0.9, 1.3)	0.7, 1.3	1.2 (0.6, 1.7)	0.6 (0.5, 0.7)
Albumin quotient ^1^	4.7 (4.0, 5.4)	5.6 (4.6, 6.7)	3.8, 10.2	8.6 (3.9, 13.4)	5.9 (4.7, 7.1)
Disease duration at baseline (years) ^1^	0.1 (0.1, 0.2)	3.6 (1.6, 5.7)	17.2, 30.0	6.5 (0, 15.5)	
Disease duration at follow-up (years) ^1^	7.0 (6.3, 7.6)	12.2 (10.2, 14.3)	27.2, 37.7	13.8 (4.0, 23.6)	
Clinical follow-up time (years) ^1^	6.9 (6.2, 7.5)	8.6 (8.0, 9.2)	7.7, 10.0	7.2 (5.5, 8.9)	
Clinical diagnosis at follow-up					
CIS	8 (25%)	—	—	—	
RRMS	24 (75%)	22 (88%)	—	—	
SPMS	0 (0%)	3 (12%)	2 / 2	—	
PPMS	0 (0%)	—	—	9 (100%)	
EDSS at baseline ^2^	0.0 (0.0–2.0)	0.0 (0.0–3.5)	5.0, 4.5	4.5 (1.0–6.5)	
Progression index at baseline ^2^	NA	0.2 (0.0–1.0)	0.2, 0.3	0.7 (0.2–1.1)	
EDSS at follow-up ^2^	1.0 (0–4)	1.0 (0.0–7.0)	7.0, 6.5	6.5 (4.0–8.0)	
Progression index at follow-up ^2^	0.1 (0.0–0.4)	0.1 (0.0–0.6)	0.2, 0.3	0.5 (0.1–2.0)	
Delta EDSS BL-FU ^2^	0.0 (-1.0–4.0)	0.0 (-1.0–4.0)	2.0, 2.0	2.0 (0.0–5.5)	
EDSS progression ^2^	9 (28%)	9 (36%)	2 / 2	8 (89%)	
Number of relapses at baseline ^2^	1	2 (1–3)		0	
Number of relapses at follow-up ^2^	2 (0–8)	3 (2–11)	6	0	
Relapse rate at follow-up ^2^	0.3 (0.0–1.0)	0.4 (0.2–1.2)	0.6	0	
MRI T2 lesions at baseline ^2^	7 (0–30)	10 (0–35)	37	14 (6–28)	
≥9 MRI T2 lesions at baseline	12 (38%)	12 (48%)	1 / 1	7 (78%)	
MRI Gd lesions at baseline ^2^	0 (0–3)	1 (0–12)	1	0 (0–3)	

BL = baseline; CSF = cerebrospinal fluid; EDSS = Expanded Disability Status Scale; Gd = gadolinium; IgG = immunoglobulin G; MRI = magnetic resonance imaging; OCB = oligoclonal bands; OND = 10 inflammatory and 30 non-inflammatory OND; EDSS progression = delta EDSS 1 for BL EDSS 0–5.5, delta EDSS 0.5 for BL EDSS 6–10. NA = not applicable (disease duration < 1 year).

Data are shown as ^1^ means with 95% confidence intervals, ^2^ median with range or individual data for the two SPMS patients.

The control group consisted of 40 patients with other neurological diseases (OND). The OND group included 30 patients with noninflammatory OND (pseudotumor cerebri, migraine, psychogenic neurological symptoms, sinus venous thrombosis, cavernoma, vascular leukoencephalopathy, seizure, herniated vertebral disc, transient ischemic attack, multiple system atrophy, neuropathic pain syndrome, cerebellar infarction, brain tumor, focal dystonia, ischemic transverse spinal cord syndrome, phobic postural vertigo, spinocerebellar ataxia, paresis of peripheral nerves) and 10 patients with inflammatory OND (infectious myelitis, ventriculitis, viral meningoencephalitis, vasculitis, systemic lupus erythematosus, antiphospholipid syndrome, sarcoidosis, NMDA receptor encephalitis and Guillain-Barré syndrome).

### Sample collection

CSF (3–8 ml), EDTA-treated whole blood and serum samples (Sarstaedt Monovettes, Nuembrecht, Germany) were obtained once during standard diagnostic lumbar puncture and peripheral vein puncture, respectively. CSF samples were immediately analyzed for CSF cell populations within 30 minutes after lumbar puncture and cell-free supernatants were stored at -80°C for further diagnostic and scientific purposes. Serum was prepared by centrifugation, 10 min at 3000 rpm (1620 g), and stored at -20°C until analysis was performed.

CSF samples were routinely analyzed for cell counts, quantitative (IgM and IgG indices) and qualitative (oligoclonal bands) analysis of intrathecal Ig-synthesis, and CSF to serum albumin ratio (albumin-quotient) as indicators of the blood-brain barrier status immediately after lumbar puncture using standard methods (Table 1).

### Characterization of CSF cell populations by flow cytometry

Staining of CSF cells was done as described [9] with the following modifications: CSF was immediately spun down after lumbar puncture for 10 minutes at 1500 rpm (1050 g). After removing the supernatant, pellets were resuspended in 50 μl BD Cell-Wash (BD Biosciences, San Jose, CA, USA) and CSF cells were stained with fluorochrome-labeled antibodies to the following human leukocyte surface antigens (all BD Biosciences) for a total of 30 minutes at room temperature in the dark:

1. 5 μl CD-45 PerCP (BD 345809), 10 μl CD19-FITC (BD 245776) and 10 μl CD138- PE (BD 347192).
2. 10 μl TriTest CD45- PerCP/CD3-FITC/CD19-PE (BD 342412).

Only when enough CSF cells were available stainings for monocytes (5 μl CD-45 PerCP, BD 345809; 5 μl CD14-FITC, BD 345784; 5 μl HLA-DR-PE, BD 340689), natural killer cells (10 μl TriTest CD45-PerCP/CD3-FITC/ CD16+56-PE, BD 342411) and memory B cells (5 μl CD-45 PerCP, BD 345809; 10 μl CD27- FITC, BD 555440; 10 μl CD19-PE, BD 345777) were also included.

Erythrocytes were lysed for 10 minutes using 2 ml of lysing solution (BD Biosciences) according to manufacturer’s protocol. Then, the tubes were centrifuged and the supernatants discarded. After one additional washing step with 2 ml BD Cell-Wash, CSF lymphocyte subpopulations were analyzed using three-color flow cytometry on a BD FACScan with Cell Quest software (BD Biosciences). Lymphocytes and monocytes were gated according to their forward (FSC) and side scatter (SSC) properties. A minimum number of 1000 events for each CSF staining were acquired for analysis with lyse-wash instrument settings, threshold FL-3 (PerCP) channel at 300 and the gate adjusted SSC versus FL- 3. Cell populations are shown in % of total lymphocytes.

### MRI protocol

All MS patients were examined by a standardized brain MRI protocol before diagnostic lumbar puncture. Patients were scanned on a 1.5 T whole-body MR scanner (Magnetom Avanto, Siemens, Germany) using the following protocol:

1. Coronal MPRage (repetition time (TR) 1600 ms, Inversion time (TI) 800 ms, echotime (TE) 3.44 ms, matrix 256x224, field of view 230x193 mm, slice thickness of 1.2 mm, number of excitations 1, iPAT factor 2)
2. Transversal diffusion tensor imaging by using a EPI sequence (TR 6000 ms, TE 94 ms, matrix 128x128 interpolated to 256x256, field of view 220x220 mm, slice thickness of 3 mm, 35 slices, iPAT factor 2, diffusion- sensitizing gradients in six directions)
3. Sagittal SPACE 3D with darkfluid preparation (TR 6000 ms, TI 2200 ms, TE 328 ms, matrix 256x236, field of view 240x221 mm, slice thickness 0.9 mm)
4. Pause of at least five minutes after administration of contrast agent (Gadolinium-DTPA)
5. Repetition of sequence 1

### Follow up data

Patients had scheduled follow-up visits every 3–6 months at the MS center. History on any neurological symptoms, relapse(s), MS disease course, EDSS, current treatment(s) and concomitant disorders were assessed and documented. Relapses had to be confirmed [23] at the MS center by a neurologist and were treated with intravenous high-dose methylprednisolone for 3–5 days. EDSS 6 month confirmed disability progression was defined as an EDSS increase ≥1 points when baseline EDSS ranged from 0–5.5 and an EDSS increase ≥0.5 within ranges of 6–10 (“delta EDSS”). MRI follow-up was not done due to different timepoints of examinations, different raters and missing standardized rating methods. Comparing the relapsing forms of MS to PPMS and OND, the term “bout- onset MS” (CIS, RRMS and SPMS) was used. Progression index at follow-up was calculated as EDSS divided by years since baseline for comparison with the cohort of Cepok et al as described before [9]. CD19:CD14 ratio was analyzed in a subset of 41 samples for further comparisons.

### Statistical analysis

Statistical analysis (means, medians, range, standard deviations, 95% confidence intervals), significance of group differences and linear regression were evaluated using SPSS software (IBM SPSS Statistics 24.0). The distribution of lymphocytes was determined via mixed ANOVA adjusted for age, sex and leucocyte count after log-transformation of non-normal distributed data. A binary logistic regression was used to evaluate influence of different factors on disease progression and disease activity with all parameters entered at the first step. Cox regression analysis was used to evaluate influence of different factors on conversions to clinically definite MS after a CIS with all parameters entered at the first step. Correlations were analyzed using Spearman’s or Pearson’s correlation. Statistical significance was defined as two-sided p-value 0.05 and p-values were corrected for multiple comparisons using Bonferroni’s correction if appropriate.

## Results

### CSF lymphocyte populations significantly differ in MS subtypes and OND

**Fig 1:**
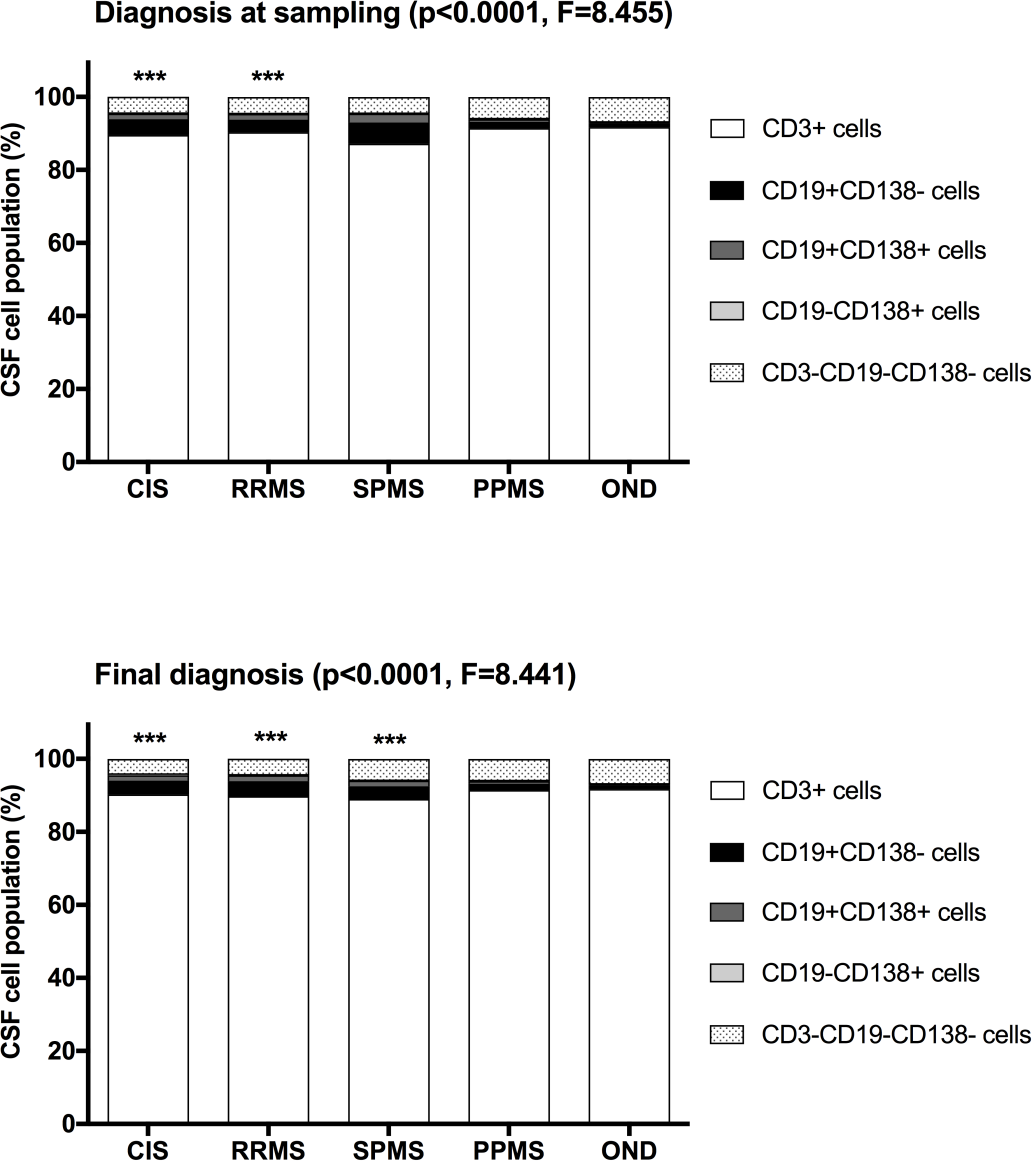
CSF lymphocyte populations in CIS, RRMS, SPMS, PPMS and OND at sampling and last follow up. Bars represent mean of individual CSF cell populations. Log-transformed data were compared using mixed (paired 2-way) ANOVA with sex, age and CSF leukocyte cell numbers as covariates to exclude confounders. *** significant difference to OND (p<0.001).

B cells were present in the CSF of almost all patients with CIS, RRMS and CPMS. They were characterized by a combination of CD19 and CD138 staining. The majority of all CSF B cells were CD19+CD138- mature B cells and CD19+CD138+ plasma blasts. A low percentage of CD19-CD138+ long-lived plasma cells (<1%) was observed in each group (Table 1). CSF lymphocyte composition was significantly different in CIS, RRMS and SPMS at follow up as compared to OND (p<0.001). CSF lymphocyte composition in PPMS and in SPMS at sampling did not significantly differ from the OND group (Fig 1). Within the OND group there were no significant differences in the CSF lymphocyte composition between inflammatory and the noninflammatory OND patients using a multivariate model corrected for CSF cell numbers, age and sex no differences (S1 Table in Supplementary Tables and Figures).

In order to exclude a possible bias also absolute numbers of lymphocyte populations were compared between patients with CIS, RRMS, SPMS, PPMS and OND using multivariate 2-way ANOVA with sex and age as covariates. Results were not different from percentages of B lymphocyte subsets (S2 Fig in Supplementary Tables and Figures).

An overall effect of aging on CSF lymphocyte populations was excluded by correlation analysis. There were only weak correlations (R<0.4) of age with CSF parameters with the exception of the albumin quotient when analyzing the total population (S2 Table in Supplementary Tables and Figures). We have also analyzed this correlations separately for the bout-onset MS and PPMS subgroups and we found a moderate effect of aging on total CSF leukocyte numbers in bout-onset MS, but not for CSF lymphocyte subsets (S3 Table in Supplementary Tables and Figures).

CSF lymphocyte composition was significantly different between bout-onset MS (CIS, RRMS and SPMS), PPMS and OND. This difference is caused by increased CD19+CD138- mature B cells, which are increased in bout-onset MS compared to PPMS (p<0.05) and OND (p<0.001) and CD19+CD138+ plasma blasts, which are increased in bout-onset MS (p<0.001) and PPMS (p<0.05) compared to OND. No difference was found for CD3+ T-cells, CD19-CD138+ plasma cells and CD3-CD19-CD138- cells (Table 2). Further, no differences in the ratio of CD19+CD138+ / CD19+CD138- lymphocyte populations between patients with CIS, RRMS, SPMS, PPMS and OND could be found (S1 Fig in Supplementary Tables and Figures).

**Table 2:** CSF lymphocyte populations in bout-onset MS, PPMS and OND.

	Bout-onset MS	PPMS	OND
Number of patients	59	9	40
CD3+ cells	89.8 (88.5, 91.1)	91.5 (88.7, 94.2)	91.7 (89.7, 93.6)
CD19+CD138- cells	4.0 (3.4, 4.6) *** +	1.7 (0.7, 2.7)	0.9 (0.7, 1.2)
CD19+CD138+ cells	1.6 (1.2, 2.0) ***	0.7 (0.3, 1.2) *	0.4 (0.2, 0.6)
CD19-CD138+ cells	0.3 (0.2, 0.3)	0.3 (0, 0.7)	0.2 (0.1, 0.4)
CD3-CD19-CD138- cells	4.4 (3.4, 5.4)	5.8 (3.5, 8.0)	6.7 (4.8, 8.6)

Bout-onset MS = CIS, RRMS and SPMS; OND = 10 inflammatory and 30 non-inflammatory OND.

CSF cell populations (% of lymphocytes) are shown as means with 95% confidence intervals. Log-transformed data were compared using mixed (paired 2-way) ANOVA with sex, age and CSF leukocyte cell numbers as covariates to exclude confounders (p<0.0001, F = 16.809).

*** significant difference to OND (p<0.001)

* significant difference to OND (p<0.05)

+ significant difference to PPMS (p<0.05).

There were no significant group differences for CD3+ T-cells, CD19-CD138+ plasma cells and CD3-CD19-CD138- cells.

PPMS patients displayed significantly lower prevalence of CSF CD19+CD138- mature B cells compared to the CD19+CD138- mature B cell prevalence of bout-onset MS patients. PPMS patients were significantly older at sampling than bout-onset MS patients. Sex, disease duration at sampling, follow up time, CD19+CD138+ cells (in % of lymphocytes, log-transformed), presence of CSF IgG OCB and presence of ≥9 MRI T2 lesions did not significantly differ within the groups (Table 3).

**Table 3:** Differences in CSF lymphocyte populations and clinical parameters between bout-onset MS (CIS, RRMS and SPMS) versus PPMS.

	Bout-onset MS	PPMS	P-value	Odds ratio (95% CI)
Number of patients	59	9		
Females	41 (70%)	4 (44%)	0.272	15.0 (0.1, >100)
Age at sampling (years) ^1^	30.8 (28.1, 33.5)	50.7 (44.8, 56.6)	0.015	1.4 (1.1, 1.9)
Disease duration at sampling (years) ^1^	2.4 (0.9, 3.9)	6.5 (0.0, 15.5)	0.390	0.9 (0.7, 1.2)
Follow-up (years) ^1^	7.7 (7.2, 8.2)	7.2 (5.5, 8.9)	0.251	0.5 (0.2, 1.6)
CSF CD19+CD138- cells (%) ^1^	4.0 (3.4, 4.6)	1.7 (0.7, 2.7)	0.040	0.0 (0.0, 0.7)
CSF CD19+CD138+ cells (%) ^1^	1.6 (1.2, 2.0)	0.7 (0.3, 1.2)	0.800	0.7 (0.0, 12.8)
CSF IgG OCB	56 (95%)	8 (89%)	0.874	1.9 (0.0, >100)
≥9 MRI T2 lesions	25 (42%)	7 (78%)	0.744	1.8 (0.1, 62.2)

^1^ means with 95% confidence intervals.

Groups were compared using binary logistic regression analysis with all variables (enter model). Variable(s) entered on step 1: Sex, age at sampling (years), disease duration at sampling (years), follow-up time (years), CD19+CD138- cells (in % of lymphocytes, log-transformed), CD19+CD138+ cells (in % of lymphocytes, log-transformed), presence of CSF OCB and presence of ≥9 MRI T2 lesions. Note: R^2^ = 0.450 (Cox & Snell), 0.830 (Nagelkerke), Model Chi-square = 40.7, p<0.0001.

### Age at sampling is prognostic for disease progression

Comparing the progressive MS subtypes (SPMS, PPMS) with CIS and RRMS, the only significant difference regards age at sampling (Table 4). Chronic progressive MS patients are significantly older than CIS and relapsing remitting MS patients. No significant difference could be found in sex, disease duration at sampling (years), follow-up time (years), CD19+CD138- cells, CD19+CD138+ cells, presence of CSF IgG OCB and presence of ≥9 MRI T2 lesions. Moreover, no significant differences were found in a subgroup analysis for bout-onset patients.

**Table 4:** Differences in CSF lymphocyte populations and clinical parameters between MS patients with (SPMS and PPMS) and without disease progression (CIS, RRMS).

	No progression (CIS, RRMS)	Progression (SPMS, PPMS)	P-value	Odds ratio (95% CI)
Number of patients	54	14		
Females	39 (72%)	6 (43%)	0.380	0.3 (0.0, 3.8)
Age at sampling (years) ^1^	29.3 (26.9, 31.7)	49.4 (44.0, 54.7)	0.041	1.7 (1.0, 3.0)
Disease duration at sampling (years) ^1^	1.7 (0.6, 2.7)	7.8 (1.0, 14.7)	0.097	1.3 (0.9, 1.9)
Follow-up (years) ^1^	7.5 (7.0, 8.0)	8.0 (6.7, 9.2)	0.441	1.4 (0.6, 3.1)
CSF CD19+CD138- cells (%) ^1^	4.0 (3.4, 4.7)	2.3 (1.3, 3.4)	0.166	0.0 (0.0, 7.8)
CSF CD19+CD138+ cells (%) ^1^	1.6 (1.2, 2.0)	1.0 (0.6, 1.5)	0.736	0.5 (0.0, 26.3)
CSF IgG OCB	51 (94%)	13 (93%)	0.156	231 (0.1, >1000)
≥9 MRI T2 lesions	23 (43%)	9 (64%)	0.657	1.9 (0.1, 31.9)

^1^ means with 95% confidence intervals.

Groups were compared using binary logistic regression analysis with all variables (enter model). Variable(s) entered on step 1: Sex, age at sampling (years), disease duration at sampling (years), follow-up time (years), CD19+CD138- cells (in % of lymphocytes, log-transformed), CD19+CD138+ cells (in % of lymphocytes, log-transformed), presence of CSF OCB and presence of ≥9 MRI T2 lesions. Note: R^2^ = 0.521 (Cox & Snell), 0.817 (Nagelkerke), Model Chi-square = 50.1, p<0.0001.

Age at sampling was also the only significant predictor for EDSS progression with higher age associated with EDSS progression (Table 5), whereas no significant differences could be found for sex, disease duration at sampling, follow-up time, CD19+CD138- cells, CD19+CD138+ cells, presence of CSF IgG OCB and presence of ≥9 MRI T2 lesions. Again, no significant differences were found for bout-onset patients.

**Table 5:** Differences in lymphocyte populations and clinical parameters between MS patients with and without EDSS progression (delta EDSS 1 for BL EDSS 0–5.5, delta EDSS 0.5 for BL EDSS 6–10).

	No EDSS progression	EDSS progression	P-value	Odds ratio (95% CI)
Number of patients	40	28		
Females	27 (67%)	18 (64%)	0.638	1.4 (0.4, 4.9)
Age at sampling (years) ^1^	30.0 (26.5, 33.4)	38.4 (33.7, 43.1)	0.012	1.1 (1.0, 1.1)
Disease duration at sampling (years) ^1^	2.8 (0.6, 5.0)	3.1 (0.6, 5.7)	0.151	0.9 (0.8, 1.0)
Follow-up (years) ^1^	7.6 (7.0, 8.1)	7.7 (6.9, 8.5)	0.921	1.0 (0.7, 1.3)
CSF CD19+CD138- cells (%) ^1^	4.2 (3.4, 5.0)	2.9 (2.2, 3.6)	0.077	0.1 (0.0, 1.2)
CSF CD19+CD138+ cells (%) ^1^	1.7 (1.1, 2.3)	1.2 (0.8, 1.6)	0.531	1.6 (0.4, 6.7)
CSF IgG OCB	39 (97%)	25 (89%)	0.303	0.2 (0.0, 3.5)
≥9 MRI T2 lesions	18 (45%)	14 (50%)	0.984	1.0 (0.3, 3.1)

^1^ means with 95% confidence intervals.

Groups were compared using binary logistic regression analysis with all variables (enter model). Variable(s) entered on step 1: Sex, age at sampling (years), disease duration at sampling (years), follow-up time (years), CD19+CD138- cells (in % of lymphocytes, log-transformed), CD19+CD138+ cells (in % of lymphocytes, log-transformed), presence of CSF OCB and presence of ≥9 MRI T2 lesions. Note: R^2^ = 0.205 (Cox & Snell), 0.276 (Nagelkerke), Model Chi-square = 15.6, p = 0.049.

### Age and MRI T2 lesions predict conversion to RRMS after CIS

24 of 32 (75%) patients with a CIS converted to clinically definite MS during the mean follow-up period of 6.9 years. A cox regression analysis was performed to identify independent predictors for RRMS conversion in the CIS cohort. Younger age at onset and the presence of ≥9 MRI T2 lesions were significant predictors of conversion to RRMS, whereas no effect was found for sex, disease duration at sampling, follow-up time, CD19+CD138- cells, CD19+CD138+ cells and presence of CSF OCB (Table 6).

**Table 6:** CSF lymphocyte populations and clinical parameters of CIS patients with and without conversion to RRMS.

	CIS	Conversion to RRMS	P-value	Odds ratio (95% CI)
Number of patients	8	24		
Time to second relapse (years) ^1^		2.5 (1.5, 3.4)		
Females	4 (50%)	18 (75%)	0.316	1.8 (0.6–5.8)
Age at sampling (years) ^1^	34.6 (25.8, 43.4)	25.4 (22.4, 28.4)	0.009	0.9 (0.9–1.0)
Follow-up (years) ^1^	6.3 (4.9, 7.7)	7.1 (6.3, 7.9)	0.578	1.1 (0.8–1.4)
CSF CD19+CD138- cells (%) ^1^	3.8 (2.8, 4.8)	4.5 (3.3, 5.8)	0.555	0.5 (0.1–4.4)
CSF CD19+CD138+ cells (%) ^1^	1.5 (0.6, 2.4)	1.6 (0.9, 2.3)	0.420	1.7 (0.5–6.0)
CSF IgG OCB	8 (100%)	22 (92%)	0.731	0.7 (0.1–5.4)
≥9 MRI T2 lesions	2 (25%)	10 (42%)	0.045	2.9 (1.0–8.5)

^1^ means with 95% confidence intervals.

Groups were compared using cox regression analysis with all variables (enter model). Variable(s) entered on step 1: Sex, age at sampling (years), disease duration at sampling (years), follow-up time (years), CD19+CD138- cells (in % of lymphocytes, log-transformed), CD19+CD138+ cells (in % of lymphocytes, log-transformed), presence of CSF OCB and presence of ≥9 MRI T2 lesions.

### B-cell/ monocyte ratio is not prognostic for disease course

Since a previous study suggested an association of the CD19:CD14 ratio with disease progression [9], the CD19:CD14 ratio was analyzed in a subset of 41 samples as described in the methods part. There was a significant correlation of the CD19:CD14 ratio with CSF leukocytes numbers (Spearman R = 0.599, corrected p-value = 0.001) and IgG Index (Spearman R = 0.487, corrected p-value = 0.020). No significant correlations were found for age at sampling, disease duration at sampling, Albumin quotient, MRI T2 lesions at sampling, MRI Gd+ lesions at sampling, EDSS at sampling and follow up, progression index at baseline and follow up, EDSS progression, number of relapses at sampling and follow up and relapse rate at follow up.

## Discussion

This prospective study was performed to determine whether different B cell subsets have predictive value in the prognosis of MS as measured by disease progression, EDSS progression and conversion from CIS to CDMS.

Main findings from our previous study [4] and baseline data from the further recruited patients demonstrated that the majority of the CIS and RRMS patients have elevated CSF B cell levels compared to progressive MS and OND. The most frequent B cell population were CD19+CD138- mature B cells followed by CD19+CD138+ plasma blasts.

We previously demonstrated a correlation of CSF B cell levels with MRI T2 lesion number, the presence of Gd-enhancing MRI lesions, total number of CSF cells, intrathecal IgM and IgG synthesis, intrathecal MMP-9 and CxCL-13 production [4]. A correlation of lesion activity on MRI with the number of plasma blasts and total CD19+ B cell numbers was also seen in other studies (16).

To our best knowledge there are no studies measuring the clinical outcome of MS patients after analysis of B cell subsets in a prospective longitudinal setting. Previous studies showed that OCB negative patients have a decreased immune response defined by less disability progression and risk of conversion to SPMS [24]. Oligoclonal IgM bands were predictive for disability progression and relapse activity [25,26]. These findings suggest a role of B cell numbers in the disease course of MS patients. In order to find a more sensitive prognostic marker for CSF inflammatory activity, we analyzed the possible association of CSF B cell populations with disease and EDSS progression and conversion from CIS to CDMS. B cell phenotypes presented in a different composition depending on the MS subtype, they were not prognostic for further disease course. Mature B cell levels in patients with EDSS progression were lower but these results were not statistically significant. This could be due to our relatively small sample size and needs further investigation.

PPMS patients were significantly older at baseline than bout onset MS patients. This fact is well known [27]. Other parameters did not show prognostic value in our follow up. The only significant difference comparing progressive (SPMS, PPMS) with the non-progressive subtypes (CIS, RRMS) and EDSS progression with the non EDSS progressing subgroup was patient age at baseline. This was already well described for PPMS [27] and underlines the importance of age correction in the analysis of CSF samples in different MS subtypes.

High CSF B-cell/monocyte ratio was reported to correlate with rapid disease progression and a higher individual EDSS progression index [9]. CSF B-cell/ monocyte ratio and T-cell/ monocyte ratios appeared to be strong indicators of rapid progression (<22 month) from CIS to CDMS [8]. In the study of Cepok et al the B-cell/ monocyte ratio showed a positive correlation with the individual disease progression of patients which has been defined as EDSS score divided by the number of years since the occurrence of the first neurological symptoms [9]. In our study, B cell/ monocyte ratio was analyzed in 41 of our samples. Only 9 of our patients were comparable with this cohort [9] with a previous disease course of at least 3 years. We did not find a significant correlation in any of our outcome variables in this comparison.

We found significantly different CSF lymphocyte compositions in CIS, RRMS and SPMS as compared to OND. CSF lymphocyte composition in PPMS did not significantly differ from the OND group. The prevalence of CD19+CD138- mature B cells was higher in bout-onset MS compared to PPMS and OND and the prevalence of CD19+CD138+ plasma blasts was higher in bout-onset MS and PPMS compared to OND. Despite this finding, inflammatory markers such as intrathecal IgG synthesis and elevated leukocyte cell count were found in both, the bout onset MS and the PPMS patients. We found a fundamental difference in the CSF lymphocyte populations in the PPMS patients. While PPMS is characterized by a distinctively different clinical course compared to bout onset MS, pathological processes like inflammation, remyelination, neurodegeneration and glial scar formation are present in all subtypes [28]. B cells are present in both, the perivascular space and the parenchyma and persist in the CSF and CNS. They may form follicles within meninges in SPMS. This finding was associated with focal cortical pathology [7]. The inflammation is present in both, bout onset MS and PPMS but the complex mechanism of disease activity and progression is not completely understood. Progressive MS may involve diffuse neurodegenerative processes which take place behind the blood brain barrier and involve microglial activation, altered axonal iron homeostasis and mitochondrial injury [29,30]. Demyelinating inflammatory activity appears to be microcompartimentalized rather than systemic and appears to be present in different intensities. Our findings support the concept of CD20 antibody targeted therapy not only in bout onset MS [11,31–34], but also in PPMS [12] considering the increased levels of plasma blasts (CD19+CD138+) in the CSF of PPMS patients which express CD20 [7] and may play a role in the inflammatory process in PPMS as evidenced by the recently reported efficacy of Ocrelizumab in PPMS [12].

Finally, our results confirm that age and MRI T2 lesion load predict conversion to RRMS after a first demyelinating event. Lower age and presence of ≥9 MRI T2 lesions were associated with a significantly higher risk of conversion to CDMS in our study, we observed similar trends for predictive data as demonstrated in other studies, in which these did reach statistical significance in all parameters [35], for increasing numbers of T2 lesions to be predictive for CIS conversion [36,37] and age, which was inversely associated to be predictive for conversion in other studies [38]. In contrast to previous studies, presence of OCB was not significantly associated with conversion to RRMS in our study [39–42]. As previously published we did not find a significant correlation between sex and risk of conversion [35,43–45]. Compared to other studies, our CIS population with available follow up data was at a lower number of patients available (N = 32).

Our study has the following limitations: due to technical reasons in this longterm setting we focused on only 3 parameters to analyze the outcome of our patients. No patient had an annualized relapse rate considered as a “high relapse rate” (>1.5) in our study and no correlation between relapse rate and B cell counts could be found. This is especially limiting for the prognostic value of our baseline results, which we were aiming for in this prospective study. A correlation of intrathecal B cells with relapse activity was hardly detectable in other studies [8,25] and the relapse rates in those studies were comparable with our MS cohort. Nearly all of our patients with bout onset MS received a disease modifying therapy but we did not consider the type or duration of this therapy for our analysis. Serial lumbar punctures were not performed in our analysis. Results from other investigations showed that the disease duration did not affect the number of CSF plasma blasts and that the proportion within the B cells was stable at different stages of disease in measurements from serial lumbar punctures [16].

## Conclusion

We detected significant differences in the CSF B cell subsets between MS subtypes and OND patients, but CSF B cells had no predictive role for disease and EDSS progression or conversion to CDMS after a CIS. Age was the only predictive variable for EDSS progression, which underlines the importance of age correction in the analysis of CSF samples in different MS subtypes. We aim to further investigate the potential prognostic value of B cells in future studies with larger patient cohort.

## Supporting information

S1 Table Differences in lymphocyte populations and cerebrospinal fluid parameters between patients with other inflammatory and noninflammatory neurological diseases. ^1^ means with 95% confidence intervals. For the univariate analysis groups were compared using ^1^ Fisher’s exact test and ^2^ independent samples Student’s t-test and ^3^ independent samples Student’s t-test with log-transformed data. ^4^ log-transformed data were compared using multivariate 2-way ANOVA with sex, age and CSF leukocyte cell numbers as covariates, exclude confounders.
(PDF)

S2 Table Correlation of age at sampling with CSF parameters. (PDF)

S3 Table Correlation of age at sampling with CSF parameters within MS subgroups. * correlation is significant at the 0.05 level (2-tailed, corrected for 2 comparisons), ** correlation is significant at the 0.01 level (2-tailed, corrected for 2 comparisons).
(PDF)

S1 Fig Differences in the ratio of CD19+CD138+ / CD19+CD138- lymphocyte populations between patients with with CIS, RRMS, SPMS, PPMS and OND. Individual data points are shown as open circles and means as grey bars. Log-transformed data were compared using univariate ANOVA with sex and age as covariates, exclude confounders. The overall p-value is indicated in the graph.
(PDF)

S2 Fig Differences in absolute numbers of lymphocyte populations between patients with CIS, RRMS, SPMS, PPMS and OND at sampling. Individual data points are shown as open circles and means as grey bars. The total numbers of lymphocyte populations were calculated from total number of cerebrospinal fluid (CSF) leukocytes and the percentage of lymphocytes with CSF leukocytes. Log-transformed data were compared using multivariate 2-way ANOVA with sex and age as covariates, exclude confounders. The overall p-values are indicated in each graph and brackets indicate significant differences between groups at p<0.05 (*) or p<0.001 (***) as analyzed by Bonferroni’s post-hoc test.
(PDF)
