# Supplementary material for: Cerebrospinal fluid B cells and disease progression in multiple sclerosis - A longitudinal prospective study
Source: PLoS One. 2017 Aug 4;12(8):e0182462. doi: 10.1371/journal.pone.0182462 (PMC5544180; doi:10.1371/journal.pone.0182462)
Supplement: S1 Table — 1 means with 95% confidence intervals. For the univariate analysis groups were compared using 1 Fisher’s exact test and 2 independent samples Student’s t-test and 3 independent samples Student’s t-test with log-transformed data. 4 log-transformed data were compared using multivariate 2-way ANOVA with sex, age and CSF leukocyte cell numbers as covariates, exclude confounders. (PDF) [file pone.0182462.s001.pdf]

PONE-D-17-15170

Cerebrospinal fluid B cells and disease progression in multiple sclerosis - A longitudinal prospective study

Supporting Information

Supplementary Table

S1 Table. Differences in lymphocyte populations and cerebrospinal fluid parameters between patients with other inflammatory and noninflammatory neurological diseases.

	Other inflammatory neurological diseases	Other non-inflammatory neurological diseases	P-value Univariate analysis	P-value Multivariate analysis
Number of patients	10	30		
Females	6 (60%)	20 (67%)	0.718 [1]	
Age at sampling (years) [1]	34.7 (33.4, 46.1)	39.4 (33.6, 45.3)	0.420 [2]	
CSF leukocytes / µl [1]	44.4 (0.0, 97.6)	2.7 (0.9, 4.6)	0.002 [3]	
CSF IgG index [1]	1.00 (0.54, 1.47)	0.51 (0.47, 0.55)	<0.001 [3]	
CSF albumin quotient [1]	5.78 (2.30, 9.25))	5.95 (4.65, 7.25)	0.905 [3]	
CSF IgG oligoclonal bands	3/9 (33%)	2/27 (7%)	0.088 [1]	
CSF CD3+ cells [1]	92.5 (88.8, 96.2)	91.4 (89.1, 93.6)	0.655 [3]	0.789 [4]
CSF CD19+CD138- cells (%) [1]	1.58 (0.81, 2.36)	0.73 (0.54, 0.92)	0.012 [3]	0.219 [4]
CSF CD19+CD138+ cells (%) [1]	0.73 (0.09, 1.36)	0.29 (0.16, 0.42)	0.082 [3]	0.198 [4]
CSF CD19-CD138+ cells (%) [1]	0.55 (0.04, 1.15)	0.15 (0.09, 0.21)	0.004 [3]	0.099 [4]
CSF CD3-CD19-CD138- cells (%) [1]	4.67 (1.96, 7.37)	7.39 (4.94, 9.84)	0.104 [3]	0.709 [4]

[1] means with 95% confidence intervals. For the univariate analysis groups were compared using [1] Fisher's exact test and [2] independent samples Student's t-test and [3] independent samples Student's t-test with log-transformed data. [4] log-transformed data were compared using multivariate 2-way ANOVA with sex, age and CSF leukocyte cell numbers as covariates, exclude confounders.
