# Supplementary material for: Cerebrospinal fluid B cells and disease progression in multiple sclerosis - A longitudinal prospective study
Source: PLoS One. 2017 Aug 4;12(8):e0182462. doi: 10.1371/journal.pone.0182462 (PMC5544180; doi:10.1371/journal.pone.0182462)
Supplement: S2 Table — (PDF) [file pone.0182462.s002.pdf]

PONE-D-17-15170

Cerebrospinal fluid B cells and disease progression in multiple sclerosis - A longitudinal prospective study

Supporting Information

Supplementary Table

S2 Table. Correlation of age at sampling with CSF parameters.

	Pearson's correlation				Spearman's correlation		
**Age at sampling versus**	**R**	**95% CI**	**R squared**	**P-value**	**Rho**	**95% CI**	**P-value**
CSF leukocytes / µl	0.044	-0.146, 0.231	0.002	0.649	-0.228	-0.404, -0.035	0.018
CSF erythrocytes / µl	-0.117	-0.299, 0.074	0.013	0.229	0.023	-0.173, 0.216	0.816
IgG index	-0.221	-0.396, -0.032	0.049	0.022	-0.247	-0.423, -0.054	0.011
Albumin quotient	0.453	0.288, 0.592	0.205	<0.0001	0.393	0.214, 0.547	<0.0001
CSF CD3+ cells (%)	0.068	-0.123, 0.253	0.005	0.487	0.084	-0.112, 0.274	0.386
CSF CD19+CD138- cells (%)	-0.212	-0.386, -0.0249	0.045	0.027	-0.184	-0.365, 0.011	0.056
CSF CD19+CD138+ cells (%)	-0.191	-0.367, -0.003	0.037	0.047	-0.145	-0.330, 0.051	0.134
CSF CD19-CD138+ cells (%)	-0.242	-0.413, -0.056	0.059	0.011	-0.248	-0.422, -0.056	0.010
CSF CD3-CD19-CD138- cells (%)	0.095	-0.095, 0.279	0.009	0.326	0.128	-0.068, 0.315	0.186
