# Supplementary material for: Cerebrospinal fluid B cells and disease progression in multiple sclerosis - A longitudinal prospective study
Source: PLoS One. 2017 Aug 4;12(8):e0182462. doi: 10.1371/journal.pone.0182462 (PMC5544180; doi:10.1371/journal.pone.0182462)
Supplement: S3 Table — * correlation is significant at the 0.05 level (2-tailed, corrected for 2 comparisons), ** correlation is significant at the 0.01 level (2-tailed, corrected for 2 comparisons). (PDF) [file pone.0182462.s003.pdf]

PONE-D-17-15170

Cerebrospinal fluid B cells and disease progression in multiple sclerosis - A longitudinal prospective study

Supporting Information

Supplementary Table

S3 Table. Correlation of age at sampling with CSF parameters within MS subgroups.

	Pearson's correlation R		Spearman's correlation R	
**Age at sampling versus**	**Bout-onset MS**	**PPMS**	**Bout-onset MS**	**PPMS**
Number of cases	59	9	59	9
CSF leukocytes / µl	-,310*	-0,207	-,369**	-0,150
CSF erythrocytes / µl	-0,146	-0,318	0,046	-0,070
IgG index	-0,126	-0,511	-0,088	-0,600
Albumin quotient	0,273	0,208	0,208	0,600
CSF CD3+ cells (%)	0,007	-0,379	0,034	-0,633
CSF CD19+CD138- cells (%)	-0,116	0,401	-0,087	0,483
CSF CD19+CD138+ cells (%)	-0,043	-0,052	0,068	-0,117
CSF CD19-CD138+ cells (%)	-0,117	0,067	-0,148	0,686
CSF CD3-CD19-CD138- cells (%)	0,079	0,296	-0,010	0,567

* correlation is significant at the 0.05 level (2-tailed, corrected for 2 comparisons), ** correlation is significant at the 0.01 level (2-tailed, corrected for 2 comparisons).
