# Supplementary material for: Cerebrospinal fluid B cells and disease progression in multiple sclerosis - A longitudinal prospective study
Source: PLoS One. 2017 Aug 4;12(8):e0182462. doi: 10.1371/journal.pone.0182462 (PMC5544180; doi:10.1371/journal.pone.0182462)
Supplement: S1 Fig — Individual data points are shown as open circles and means as grey bars. Log-transformed data were compared using univariate ANOVA with sex and age as covariates, exclude confounders. The overall p-value is indicated in the graph. (PDF) [file pone.0182462.s004.pdf]

PONE-D-17-15170

Cerebrospinal fluid B cells and disease progression in multiple sclerosis - A longitudinal prospective study

Supporting Information

Supplementary Figure

S1 Fig. Differences in the ratio of CD19+CD138+ / CD19+CD138- lymphocyte populations between patients with with CIS, RRMS, SPMS, PPMS and OND.

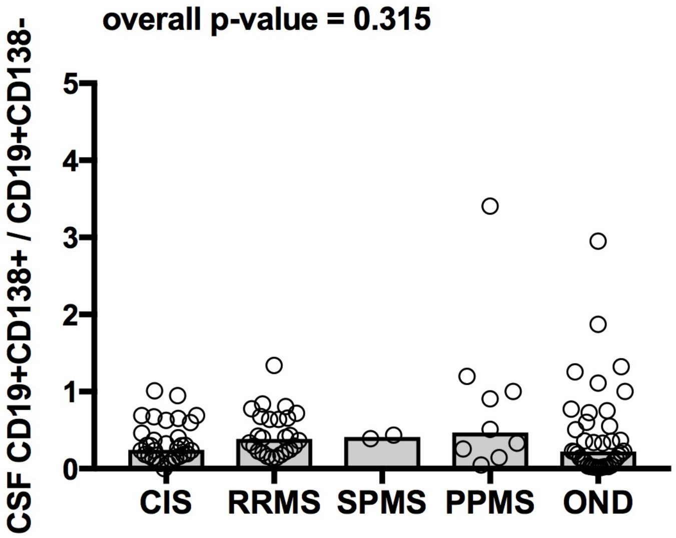

Individual data points are shown as open circles and means as grey bars. Log-transformed data were compared using univariate ANOVA with sex and age as covariates, exclude confounders. The overall p-value is indicated in the graph.
