# Supplementary material for: Cerebrospinal fluid B cells and disease progression in multiple sclerosis - A longitudinal prospective study
Source: PLoS One. 2017 Aug 4;12(8):e0182462. doi: 10.1371/journal.pone.0182462 (PMC5544180; doi:10.1371/journal.pone.0182462)
Supplement: S2 Fig — Individual data points are shown as open circles and means as grey bars. The total numbers of lymphocyte populations were calculated from total number of cerebrospinal fluid (CSF) leukocytes and the percentage of lymphocytes with CSF leukocytes. Log-transformed data were compared using multivariate 2-way ANOVA with sex and age as covariates, exclude confounders. The overall p-values are indicated in each graph and brackets indicate significant differences between groups at p<0.05 (*) or p<0.001 (***) as analyzed by Bonferroni’s post-hoc test. (PDF) [file pone.0182462.s005.pdf]

PONE-D-17-15170

Cerebrospinal fluid B cells and disease progression in multiple sclerosis - A longitudinal prospective study

Supporting Information

Supplementary Figure

S2 Fig. Differences in absolute numbers of lymphocyte populations between patients with CIS, RRMS, SPMS, PPMS and OND at sampling.

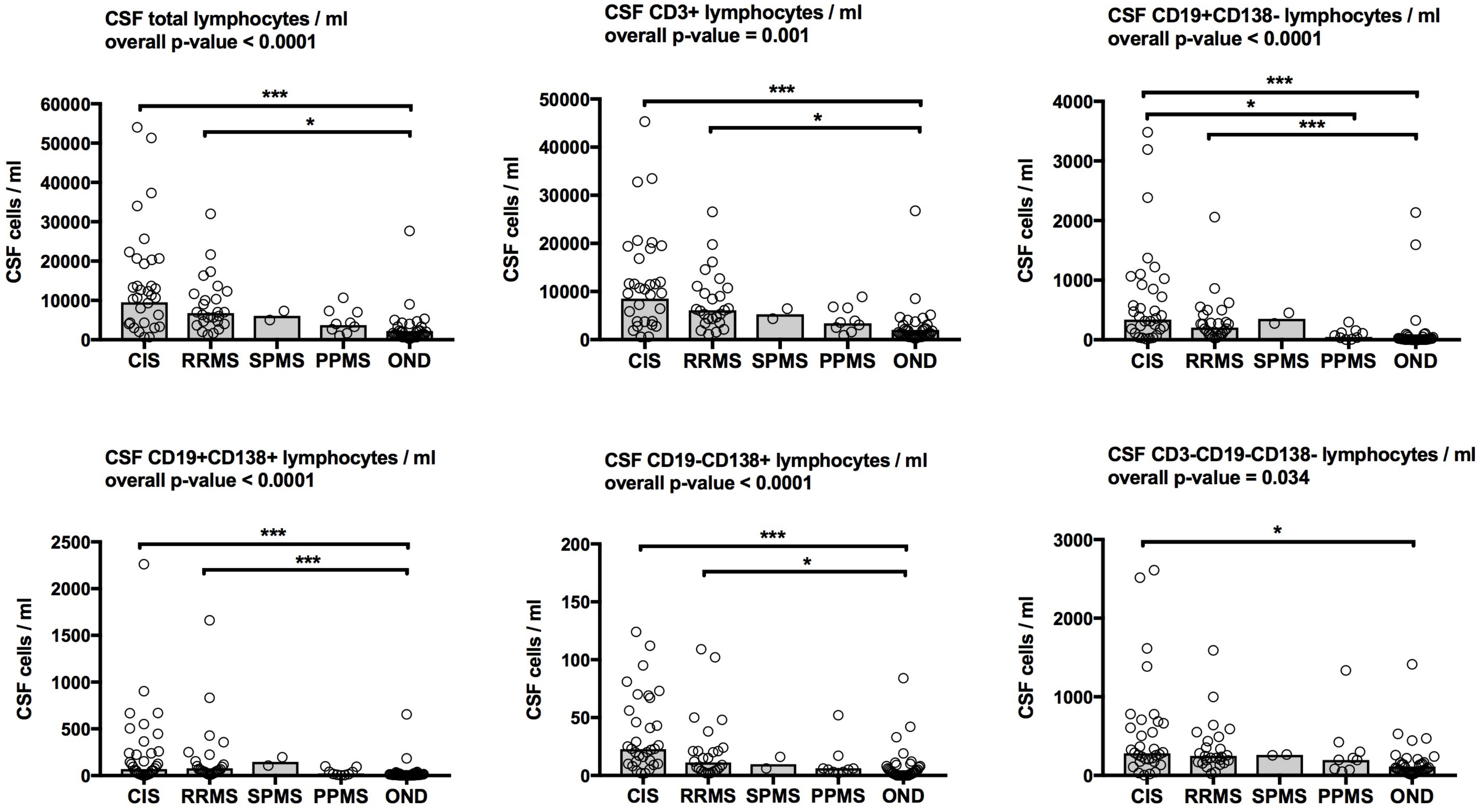

Individual data points are shown as open circles and means as grey bars. The total numbers of lymphocyte populations were calculated from total number of cerebrospinal fluid (CSF) leukocytes and the percentage of lymphocytes with CSF leukocytes. Log-transformed data were compared using multivariate 2-way ANOVA with sex and age as covariates, exclude confounders. The overall p-values are indicated in each graph and brackets indicate significant differences between groups at $p<0.05$ (*) or $p<0.001$ (***) as analyzed by Bonferroni's post-hoc test.
